# Heterogeneity in outcome assessment for inflammatory bowel disease in routine clinical practice: a mixed-methods study in a sample of English hospitals

**DOI:** 10.1136/bmjopen-2021-056413

**Published:** 2021-12-30

**Authors:** Violeta Razanskaite, Constantinos Kallis, Bridget Young, Paula R Williamson, Keith Bodger

**Affiliations:** 1 Department of Health Data Science, Institute of Population Health, University of Liverpool, Liverpool, UK; 2 Department of Public Health, Policy and Systems, Institute of Population Health, University of Liverpool, Liverpool, UK; 3 Digestive Diseases Unit, Aintree University Hospital, Liverpool, UK

**Keywords:** inflammatory bowel disease, quality in healthcare, gastroenterology

## Abstract

**Objectives:**

Knowledge of the extent of variation in outcome assessment for inflammatory bowel disease (IBD) in routine practice is limited. We aimed to describe and quantify variation in outcome coverage and to explore patient, clinician and practitioner factors associated with it.

**Design:**

Prospective exploratory mixed-methods study.

**Setting:**

IBD clinics at six hospitals in North West England with differing electronic health record (EHR) systems.

**Methods:**

Mixed-methods study comprising: (a) structured observations of outcomes elicited during consultations (102 patients consulting 24 clinicians); (b) retrospective analysis of outcomes recorded in the EHR (909 consultations; 127 clinicians) and (c) semistructured interviews with the 24 observed clinicians. We determined whether specific outcome ‘sets’ were elicited or recorded, including: (1) a minimum set of symptom pairs (‘PRO-2’); (2) symptom sets from disease activity indices and (3) a reference list of 37 symptoms, signs and impacts. Factors associated with variation were explored in univariate and multivariate binary logistic regression analyses and from clinician interviews.

**Results:**

PRO-2 coverage was not invariable (elicited during 81% of observed consultations; recorded in 56% of EHR) and infrequent for complete activity indices (all domains from Harvey-Bradshaw Index: elicited, 18%; recorded, 5%). The median number of outcomes from the reference list elicited per consultation was 12 (13-fold variation) and recorded in EHR was 7 (>20-fold variation). Symptom quantification (PRO-2) seldom adhered closely to standardised descriptors and an explicit timeframe was defined rarely. PRO-2 recording in EHR was associated with a diagnosis of ulcerative colitis (OR: 2.09 (95% CI 1.15 to 3.80)) and nurse-led consultations (OR: 6.98 (95% CI 3.28 to 14.83)) and a three-way model suggested 26% of total variability lay between clinicians, 17% between patients but the remainder was unexplained. Most clinicians expressed preference for individualised health status evaluations versus standardised outcome assessments.

**Conclusions:**

There was little evidence for standardised assessment and recording of IBD outcomes and substantial intra-clinician and inter-clinician variation from one consultation to another. Nurses demonstrated a greater tendency to standardised practice.

**Strengths and limitations of this study**

- Our work provides a unique insight into variation in day-to-day assessment and recording of clinical outcomes during inflammatory bowel disease care delivery in England.
- In view of the magnitude of variation and lack of standardisation demonstrated by our study, there is an unmet need for evidence-based guidance, policies, education and quality standards to define and address unwarranted heterogeneity in outcome assessment.
- There are limitations to using quantitative methods to define the complex range of patient, practitioner and hospital factors that may be associated with variation in outcome coverage, thus parallel interviews were undertaken to explore practitioners’ views on outcome assessment.
- Observations were conducted at six sites within one region of England and findings cannot be generalised to the whole country.

## Introduction

Disease activity in inflammatory bowel disease (IBD) may be assessed from symptoms, physical signs and laboratory, endoscopic or radiological measures of inflammation.^1^ Subjective symptoms overlap with other conditions and are neither sensitive nor specific for active bowel inflammation.^2^ However, more objective measures of intestinal inflammation are invasive, costly and may not be available to clinicians when making treatment decisions.

Clinical trials have traditionally used physician-reported composite outcome measures. These ‘activity indices’ combine symptoms, signs and some objective parameters, such as the Crohn’s Disease Activity Index (CDAI)^3^ or Mayo Score.^4^ However, the precise choice of index has varied across trials.^5–7^ Furthermore, it is recognised that the requirement for objective test results makes such instruments impractical for routine use at every clinical encounter.

Several international efforts are seeking better consensus on standardisation of outcome assessment for comparative effectiveness research, including development of core outcome sets (COSs).^8 9^ In trials, the use of symptom-based end-points as co-primary outcomes alongside more objective measures of inflammation has been proposed.^10–12^ For the symptom component, there has been the suggestion of adopting simple two-item instruments that aim to capture the dominant symptom ‘pair’ for each condition, along with a shift towards collecting such outcome data directly from patients (‘PRO-2’). The PRO-2 symptom pairs focus on stool frequency and blood in stool for ulcerative colitis (UC)^13^ and abdominal pain and stool frequency in Crohn’s disease (CD).^14^ In parallel with work focused on trials, a recent initiative has sought to promote standardisation of outcome assessment for clinical practice to support international benchmarking.^15 16^

Despite a renewed focus on standardisation, there are currently no explicit minimum data standards for IBD outcome assessment in routine settings, nor any regulatory requirement to record certain outcomes or to use specific tools. Units providing IBD care in the UK are not formally accredited at present. However, key performance indicators in the national audit of biological therapies have long encouraged the collection of traditional disease activity indices (eg, Harvey-Bradshaw Index (HBI) for CD^17^ or Simple Clinical Colitis Activity Index (SCCAI) for UC),^18^ although participation is voluntary.^19^ UK hospitals still vary widely in digital maturity and choice of electronic health record (EHR) systems which may serve as a barrier to implementing standardised outcome sets for specific conditions.

Remarkably little is known about how clinicians currently elicit and record clinical outcomes in daily IBD practice, including whether minimum ‘sets’ of core symptoms are captured systematically at each consultation. We propose that it is not unreasonable to expect that all clinical encounters might elicit and record the presence or absence of the ‘PRO-2’ symptoms and yet it is currently unknown whether such minimum symptom sets are covered invariably in routine settings. Nor do we know the extent to which practitioners vary in their approach to quantifying individual outcomes or in their coverage of broader sets of IBD outcomes such as those from traditional disease activity indices or quality of life instruments.

We hypothesised that marked variation and inconsistency will exist in the eliciting and recording of IBD outcomes. Undue variation in practice may lead to inequalities in care, including differences in thresholds for starting new treatments or in judging their effectiveness. We aimed to describe and quantify this variation in outcome coverage during routine care delivery and examine factors associated with it.

## Methods

### Study overview

We undertook an exploratory mixed-methods project involving quantitative and qualitative methods at a sample of English hospitals. This included:

1. Structured observation and audio recording of ‘live’ consultations to investigate outcomes ‘elicited’ during face-to-face interactions.
2. Retrospective review of EHRs to examine how those outcomes are ‘recorded’ by healthcare professionals.
3. Qualitative observations and interviews with clinicians to explore their approach to outcome assessment and their views on outcome standardisation.

### Selection of hospital sites, clinicians and patients

#### Hospitals

Six acute hospitals in North West England were selected purposively, reflecting a range of service sizes, digital maturity and different EHR systems, in order to explore the capture of clinical outcomes across varied Information Technology (IT) infrastructures.^20^ At each site, we determined whether the system contained pre-defined fields for capturing symptom checklists, indices, scores or other outcomes (as opposed to recording such information as unstructured text).

#### Clinicians

Participants were recruited from those delivering care to adult patients with IBD and focused on medically trained physicians and IBD specialist nurses. Clinicians were selected purposively by a local study collaborator at each site, aiming for a minimum of three clinicians per service. Of 25 clinicians invited to participate, 24 agreed. Written, informed consent was obtained. Participants comprised 10 consultant gastroenterologists, 4 gastroenterology specialist trainees and 10 specialist nurses (3–6 clinicians per hospital). The clinicians understood that the research was focused on information elicited and recorded but were not aware of the specific focus on standardised outcome assessment. Sample size calculations could not be undertaken in advance as our project was an exploratory mixed-methods study, aiming to achieve theoretical saturation.^21^

#### Patients

Patients were recruited from among those attending face-to-face consultations with the 24 participating clinicians. We aimed for a minimum of three patients per clinician, conducting observations over the course of one or more clinics. Inclusion criteria for patients were age ≥18 years, ability to communicate in English (ie, not requiring an interpreter) and a clinical diagnosis of either CD, UC or unclassified IBD (IBD-U). Patients were selected from clinic lists, sent study information before their consultation and approached by the researcher before their appointment. Of 112 patients invited to participate, 10 (9%) declined. Patients provided written consent and none withdrew after the consultation. Electronic medical records of each participating patient were subsequently reviewed to capture demographics, clinical characteristics and outcome data.

### Structured observation and audiorecording of consultations

One observer (VR, a medical gastroenterologist in training) performed structured observations of 102 clinician–patient consultations between May 2018 and June 2019. Consultations were audiorecorded, transcribed and anonymised. A structured data template was used to guide observations, focusing on outcomes elicited and recorded from pre-specified lists (outcomes sets).

### Definition of outcome sets

In the absence of a singular, internationally agreed minimum outcome set for IBD, we defined two alternative ‘sets’ of outcomes that might be regarded as candidates for routine coverage during every clinical review. The first sets were based on symptoms pairs from the relevant PRO-2 (stool frequency and abdominal pain for CD; stool frequency and rectal bleeding for UC (or IBD-U)). The second sets were focused on the items from traditional disease activity indices used in clinical trials,^5–8^ observational studies,^22^ registries^23^ and clinical audits.^19^ For CD, we selected the CDAI and HBI, and for UC we identified the Mayo Score and SCCAI. The outcomes were categorised as either a global assessment of health status, an individual symptom or a physical sign. Our study focused on information that is elicited by clinicians from patients during consultations and so we excluded results of investigations (eg, blood tests). Within the outcome sets from each disease activity index, we also defined subsets of ‘main symptoms’ from within the complete list of all domains.

To quantify the full breadth of outcome coverage during routine practice, we generated a longer list of potential symptoms, signs and life impacts of IBD that might be actively ruled in or out during a consultation. This reference list enabled us to count the number of ‘relevant’ IBD outcomes covered in each consultation. In addition to symptom pairs and the items from activity indices, we added discrete outcomes from other clinician-reported outcomes (CLIN-ROs) or patient-reported outcomes (PROs) based on existing systematic reviews of outcomes used in clinical trials^5–7^ and from guidelines for clinical practice.^15 24 25^ Life impact outcomes were classified using a standardised outcome taxonomy proposed by the Core Outcome Measures in Effectiveness Trials collaboration.^26^ This resulted in a reference list of 37 outcomes (online supplemental information S1).

10.1136/bmjopen-2021-056413.supp1 Supplementary data

### Outcomes elicited during observed consultations

VR reviewed field notes and audiorecordings for each observed consultation to determine whether each item from our reference list was covered and whether selected symptoms were quantified using standardised descriptors. ‘Eliciting’ a symptom was defined as any explicit mention regardless of formal quantification and included whenever an item was verified as being absent (ie, was actively ‘ruled out’, such as the absence of abdominal pain).

Quantification of the PRO-2 symptom sets was assessed with reference to standardised descriptors from disease activity indices. Each item was categorised according to whether the clinician used standardised descriptors in accordance with the activity indices from which the PRO-2 was derived),^13 14^ informal descriptors (non-standardised) or none at all. We also noted whether outcomes were assessed over the relevant timeframe stipulated in the indices (eg, symptoms over the last 24 hours for HBI, or the last 3 days for SCCAI). It was also noted whether a disease activity score was calculated and recorded.

### Outcomes recorded in the EHR

For each observed consultation, VR subsequently reviewed the local EHR to locate any record of the items from the outcome sets. This included clinical letters, scanned hand-written case notes, electronic data entries and any other clinician-generated information created for the observed encounter. Documentation of coverage and quantification was noted for each relevant item. This process was repeated for up to 10 consecutive previous consultations for the same patient (depending on the number available in the EHR). The details of which clinician recorded each encounter was noted (n=127 practitioners in total).

### Measures of outcomes elicited and recorded

Table 1 summarises the measures we derived for the outcomes elicited during the 102 directly observed consultations and those recorded in 909 consultation records. In addition to coverage and quantification, we determined the extent of ‘information loss’ for the observed consultations by calculating the number of items from the reference list that were elicited during the consultation but not recorded in the EHR. We also analysed the order in which outcomes were discussed to establish whether a standardised ‘checklist’ approach was used. We defined a checklist as any list of three or more outcomes that were elicited by direct questioning in a fixed order by an individual clinician during ≥2 observed consultations for patients having the same diagnosis.

**Table 1:** List of measures derived from observed consultations (n=102) and from review of consultations recorded in the electronic health record (n=909)

Measure	Observed consultations (‘elicited’ outcomes)	Electronic health records (‘recorded’ outcomes)
Coverage of outcome sets		
Coverage of PRO-2 symptom pair*	Relevant pair of symptoms elicited or not	Relevant pair of symptoms recorded or not
Coverage of relevant symptoms or signs from a disease activity index† Main symptoms All domains	Set of outcomes from a relevant index elicited or not	Set of outcomes from a relevant index recorded or not
Breadth of outcome coverage	Total number of outcomes elicited from the list of 37 pre-specified symptoms, signs and impacts‡	Total number of outcomes recorded from the list of 37 pre-specified symptoms, signs and impacts‡
Quantification of outcomes		
Quantification of PRO-2 symptoms	Whether the specific symptom was quantified using standardised descriptors during the consultation	Whether the specific symptom was quantified in the record using standardised descriptors
Recording a score for a disease activity index	N/A	Whether a relevant score is recorded in the EHR
Recording of the information elicited during a consultation		
Information loss	N/A	Total number of outcomes elicited during the observed consultation minus the number recorded in the EHR for the same consultation (n=102 paired observations)
Using a standardised ‘check list’ during consultations		
Coverage of three or more symptoms in a standardised sequence	Any ‘checklist’ of at least three symptoms elicited using direct questions by a practitioner in two or more consultations	N/A

*PRO-2 for UC (or IBD-U) comprises stool frequency and rectal bleeding; for CD comprises abdominal pain and stool frequency.

†Indices were the Simple Clinical Colitis Activity Index (SCCAI) and Partial Mayo Score for UC, and the Harvey-Bradshaw Index (HBI) and Crohn’s Disease Activity Index (CDAI) for Crohn’s disease.

‡See online supplemental information S1 for the reference list of outcomes.

CD, Crohn’s disease; EHR, electronic health record; IBD-U, inflammatory bowel disease-unclassified; UC, ulcerative colitis.

### Determining factors associated with outcome coverage

In order to explore factors associated with coverage of minimum sets of outcomes, we defined a series of patient, practitioner and site level variables. Variables were defined *a priori* based on clinical significance and to control for potential confounders, further informed by clinician interviews. Patients with a stoma were excluded where appropriate.

### Statistics

Descriptive data for the main metrics of outcome coverage are presented as percentages of consultations and variation expressed as the range of values (low to high). We used random effects multivariable logistic regression to explore factors associated with outcome set coverage. For observed consultations, a two-level model was applied (to control for repeated consultations per clinician but with only one observation per patient). A three-level model was used for clinical records (to control for repeated records per patient and clinician). We used the random effect models to estimate the percentage of variation attributed to patient or clinician levels.

### One-to-one interviews with IBD clinicians

A single interviewer (VR) conducted face-to-face qualitative interviews with each of the 24 IBD clinicians to explore influences on their approaches to outcome assessment. Interviews were semistructured and informed by an interview guide (see online supplemental information S2). Interviews lasted 25 min on average, ranging from 17 to 35 min. They were digitally audiorecorded, transcribed and anonymised. Qualitative data analysis was iterative and ongoing throughout the study drawing on thematic approaches, aided by Nvivo software package. Our work was informed by the literature on quality and rigour in qualitative research^27^ although we recognise that procedures alone do not guarantee quality.^28^

### Integration of quantitative and qualitative data

Structured observations and interviews with clinicians were conducted in parallel, allowing for simultaneous analysis of observed practices and self-reported narratives. Interim quantitative analysis of outcomes collected during observed consultations was performed after 50 observations, informing adjustments to the interview guide for the remainder of data collection (online supplemental information S3). Retrospective review of health records was performed after completion of observations and interviews, and informed further qualitative analysis to triangulate and interpret quantitative findings.

### Patient and public involvement

Our study design was discussed with, and approved by, the IBD patient panel at the hospital sponsoring the study.

## Results

### Characteristics of participating hospitals, clinicians and patients

#### Hospitals

Centres varied widely in population size served, compliment of medical and nursing staff delivering IBD services and digital maturity of IT systems (table 2). Three sites were global digital exemplars according to the Digital Maturity Assessment for English Trusts.^20^ However, at all sites, the main consultation record was a letter to the patient’s general practitioner. Letters were semistructured but variable and none contained a pre-defined symptom checklist, nor a designated field for a disease activity index score. Documentation of individual outcomes was generally within unstructured free text narratives. At two sites, outcomes or disease activity indices could be recorded electronically in a designated part of the EHR, whereas at other centres such information might be captured in hand-written entries (scanned notes). At one site, there was an electronic system for patients to directly record outcomes via a web-based application (portal), although none of the patients studied used this system.

**Table 2:** Characteristics of participating hospital sites and their IBD services, number of directly observed consultations and number of electronic health record reviews

Hospital	A	B	C	D	E	F	Total
Scale and scope of hospital*							
Population served	350 000	220 000	400 000	360 000	750 000	445 000	N/A
Inpatient beds	789	828	855	887	857	600	N/A
Digital Maturity Assessment†							
Readiness (%)	86	99	87	70	86	57	N/A
Capabilities (%)	59	83	79	26	66	51	N/A
Infrastructure (%)	98	98	89	64	89	75	N/A
Global digital exemplar		✓	✓		✓		3
IBD services‡							
Gastroenterologists (WTE)	6	8	3	3	7	2	29
IBD nurses (WTE)	2	4	1	3	2	2	14
Administrative support (WTE)	–	1	–	1	1	1	4
Research active (trials and/or BioResource)^37^		✓		✓	✓		3
Observations of IBD consultations							
Clinicians observed	6	4	3	4	4	3	24
Consultants (doctor)	2	2	2	2	1	1	10
Specialist trainees (doctor)	2	–	–	–	2	–	4
IBD nurse specialist (nurse)	2	2	1	2	1	2	10
Patients observed	24	16	16	16	17	13	102
Review of electronic health records for observed patients							
Consultation records reviewed	211	148	144	134	165	107	909
Clinicians recording consultations	35	19	15	23	20	15	127
Consultants (doctor)	8	8	7	7	7	4	41
Specialist trainees (doctor)	21	2	2	4	8	3	40
Other grades (doctor)	–	3	1	5	2	2	13
Specialist nurses (nurse)	6	6	5	7	3	6	33

The clinicians are categorised as doctors (consultants, specialist trainees and other grades) and IBD specialist nurses.

*Source: Trust Annual Reports and Accounts 2017–2018 (available online).

†Source: NHS England (available online).^20^

‡Figures provided by sites for status of service in 2018–2019.

IBD, inflammatory bowel disease.

#### Clinicians

At least one doctor and one specialist nurse were directly observed at each site with a minimum of three consultations per practitioner (table 2). Retrospective review of the EHR for the participating patients included records created by 127 clinicians in total (at least 15 per site), covering a wide range of practitioners.

#### Patients

The sample contained equal representation with respect to sex (48% men) and diagnosis (49% CD) and a broad range of ages (18–84 years), disease classification and treatment history (table 3). Reflecting the location of the hospitals and demographics of the local population, the ethnicity of the patients was almost exclusively white (97%) and mostly living in urban areas. The mean duration of disease was 13 years and approximately one-third were on immunomodulators or biologic therapies.

**Table 3:** Demographic and clinical characteristics of participating patients (n=102) at the time of the observed consultations (‘Observed’) and for consultations reviewed in their electronic health record (‘Records’)

	CD		UC/IBD-U		Total	
	Observed, n=50	Records, n=484	Observed, n=52	Records, n=425	Observed, n=102	Records, n=909
Patient demographics						
Gender						
Male, n (%)	24 (48)		25 (48)		49 (48)	
Female, n (%)	26 (52)		27 (52)		53 (52)	
Age, mean (range), years	47 (24–75)	45 (21–75)	49 (18–84)	50 (18–84)	48 (18–84)	47 (18–84)
Clinical characteristics, n (%)						
Montreal classification						
Location/extent	L1: 21 (42)	L1: 217 (45)	E1: 13 (25)	E1: 86 (20)	N/A	N/A
	L2: 10 (20)	L2: 86 (18)	E2: 21 (40)	E2: 185 (44)		
	L3: 19 (38)	L3: 181 (37)	E3: 18 (35)	E3: 154 (36)		
	+L4: 2 (4)	+L4: 22 (4.5)				
Behaviour	B1: 24 (48)	B1: 226 (47)				
	B2: 12 (24)	B2: 123 (25)				
	B3: 10 (20)	B3: 97 (20)				
	B2/B3: 4 (8)	B2/B3: 38 (8)				
Perianal involvement	12 (24)	92 (19)			N/A	N/A
Disease duration, mean (range)	15 (1–48)	14 (0–48)	10 (0–48)	10 (0–48)	13 (0–48)	12 (0–48)
Previous IBD surgery	32 (64)	300 (62)	4 (8)	27 (6)	36 (35)	324 (36)
Stoma present	12 (24)	100 (21)	4 (8)	27 (6)	16 (16)	127 (14)
Extra-intestinal manifestations	13 (26)	126 (26)	8 (15)	62 (15)	21 (21)	188 (21)
Current medical therapy						
No regular IBD medication	10 (20)	94 (19)	9 (17)	49 (12)	19 (19)	143 (16)
5-ASA only	3 (6)	33 (7)	25 (48)	228 (54)	28 (28)	261 (29)
Corticosteroids	4 (8)	48 (10)	10 (19)	61 (14)	14 (14)	109 (12)
Immunomodulators	24 (48)	240 (50)	8 (15)	84 (20)	32 (31)	313 (34)
Anti-TNF therapies	19 (38)	154 (32)	5 (10)	47 (11)	24 (24)	201 (22)
Other biologic therapies	3 (6)	24 (5)	1 (2)	6 (1)	4 (4)	30 (3)

5-ASA, 5-aminosalicyclic acid; CD, Crohn’s disease; IBD, inflammatory bowel disease; IBD-U, inflammatory bowel disease-unclassified; TNF, Tumour necrosis factor alpha; UC, ulcerative colitis.

### Coverage of PRO-2 symptom pairs

**Figure 1:**
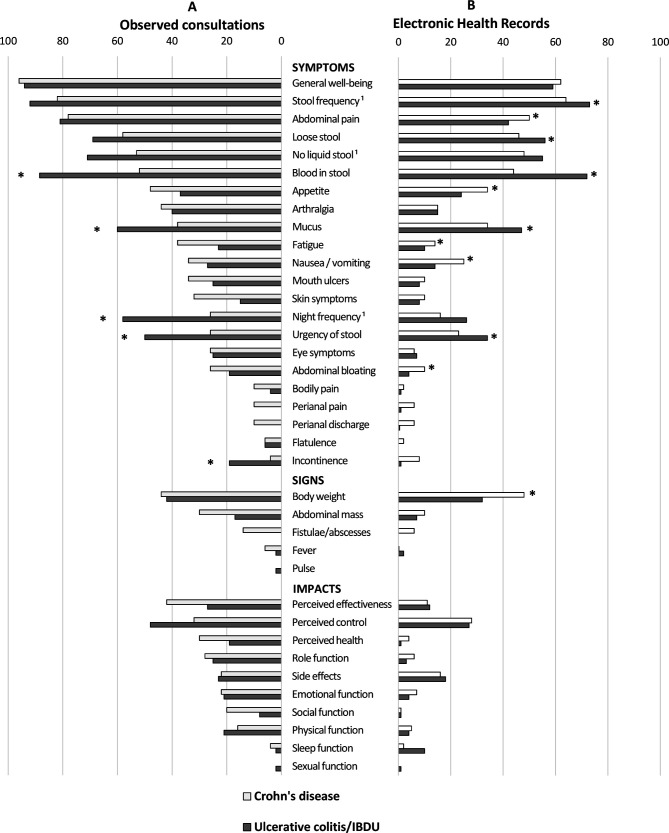
Frequency (%) of individual inflammatory bowel disease (IBD) outcomes that were: (A, left panel) elicited during observed consultations with 102 patients; or (B, right panel) recorded in the electronic health records of 909 consultations with the same patients. *Significant difference in frequency between Crohn’s disease and ulcerative colitis/IBD-U (p<0.05); ^1^Patients with a stoma excluded. IBD-U, inflammatory bowel disease-unclassified.

Analysis of the relative frequency of coverage of the 37 outcomes on our reference list confirmed that the PRO-2 symptom pairs were the ‘top two’ (most frequently) elicited and recorded gastrointestinal symptoms (figure 1). Although coverage of such symptom pairs was very common, it was not invariable in routine practice, being elicited in 81% of observed consultations but recorded in only 71% of records for those visits. In the larger scale review of records made by 127 practitioners, the presence or absence of a PRO-2 was noted in just 56% of IBD consultations (table 4).

**Table 4:** Frequency of eliciting and recording relevant PRO-2 symptom pairs for Crohn’s disease and ulcerative colitis/IBD-U during routine clinical practice at six IBD centres

	Observed consultations*	Observed consultations*	Retrospective records review†
	Elicited during visit, n (%)	Recorded in EHR, n (%)	Recorded in EHR, n (%)
All patients			
Stool frequency‡	76/86 (88)	67/86 (78)	534/782 (68)
Abdominal pain	82/102 (80)	58/102 (57)	441/909 (49)
Blood in stool	74/102 (73)	59/102 (58)	519/909 (57)
Relevant symptom pair	**70/86** (**81**)	**61/86** (**71**)	**436/782** (**56**)
Crohn’s disease			
Stool frequency‡	32/38 (84)	30/38 (79)	253/384 (66)
Abdominal pain	40/50 (80)	32/50 (64)	263/484 (54)
Both (symptom pair)	**29/38** (**76**)	**28/38** (**74**)	**182/384** (**47**)
Ulcerative colitis/IBD-U			
Stool frequency‡	44/48 (92)	37/48 (77)	281/398 (71)
Blood in stool	47/52 (90)	37/52 (71)	306/425 (72)
Both (symptom pair)	**41/48** (**85**)	**33/48** (**69**)	**254/398** (**64**)

The figures indicate whether the relevant items were ‘covered’ (actively verified as present or absent).

Values in bold indicate when both symptoms from the relevant PRO-2 were covered.

*Consultations with 102 patients by 24 practitioners.

†Including the record for the observed consultation and up to 10 consecutive previous consultations recorded in the EHR for the 102 patients by 127 practitioners.

‡Patients with a stoma excluded for analyses of stool frequency.

EHR, electronic health record; IBD, inflammatory bowel disease; IBD-U, inflammatory bowel disease-unclassified.

At the level of individual practitioners, the percentage of PRO-2 elicited during observed encounters ranged from 20% to 100% of consultations. Aggregated rates were 75% for doctors versus 94% for nurses. Eight out of 10 nurses (80%) but only 7 out of 14 doctors (50%) elicited PRO-2 in every observed consultation. Within the EHRs created for the 102 observed consultations, PRO-2 coverage ranged from 0% to 100% at practitioner level. Again, the aggregated figures suggested a systematic difference between doctors and nurses (58% vs 91%). One in five nurses (20%) recorded symptom pairs for every observed consultation, compared with one in seven doctors (14%). None of the factors studied in two-level modelling of the 102 observed consultations were significantly associated with eliciting PRO-2 but this exploratory analysis was based on low sample size (data not shown).

However, with the benefit of a large sample size of clinicians (n=127) and consultations (n=909), we were better able to explore factors associated with PRO-2 recording using a three-level model and derive estimates for the relative contributions of clinician as opposed to patient-related factors (table 5). The likelihood of finding the appropriate pair of symptoms recorded in the EHR was independently associated with a diagnosis of UC (OR: 2.09 (95% CI 1.15 to 3.80)) and nurse-led consultations (OR: 6.98 (95% CI 3.28 to 14.83)). In this three-level model (containing multiple records per patient), 26% of total variability lay between clinicians, 17% between patients and 57% of variation remained unexplained. This confirmed that clinicians from a nursing background were significantly more likely to make a formal record of a relevant symptom pair than doctors.

**Table 5:** Factors associated with recording the relevant PRO-2 symptom pair in the electronic health record (EHR) following consultations for inflammatory bowel disease

	Symptom pair recorded in the EHR			
	Univariate analysis		Multivariable analysis	
	OR (95% CI)	P value	OR (95% CI)	P value
Patient factor				
Female gender	1.47 (0.90 to 2.39)	0.123	1.41 (0.88 to 2.28)	0.155
Age	1.00 (0.98 to 1.01)	0.613	0.99 (0.97 to 1.01)	0.235
Ulcerative colitis or IBD-U	**2.54 (1.56 to 4.14**)	**<0.001**	**2.09 (1.15 to 3.80**)	**0.016**
Previous IBD surgery	0.41 (0.23 to 0.73)	0.003	0.61 (0.30 to 1.24)	0.173
Extraintestinal manifestations	1.23 (0.68 to 2.23)	0.485	1.47 (0.82 to 2.62)	0.193
Current immunosuppressive therapy	0.75 (0.48 to 1.19)	0.224	0.75 (0.46 to 1.22)	0.245
Disease duration	1.00 (0.98 to 1.02)	0.794	1.01 (0.98 to 1.03)	0.533
Practitioner factor				
Nurse consultations	**6.23 (2.91 to 13.33**)	**<0.001**	**6.98 (3.28 to 14.83**)	**<0.001**
Hospital IT factor				
Global digital exemplars	1.23 (0.55 to 2.72)	0.617	1.49 (0.71 to 3.12)	0.287

Random effects binary logistic regression models for selected patient, practitioner and site characteristics. The appropriate symptom pair for ulcerative colitis (or IBD-U) was rectal bleeding and stool frequency and for Crohn’s disease was abdominal pain and stool frequency. The likelihood of finding the appropriate symptom pair recorded was independently associated with a diagnosis of ulcerative and nurse-led visits. n=782 eligible consultation records.

Values in bold indicate factors independently associated with the recording of relevant PRO-2 in the multivariable analysis (p<0.05)

IBD, inflammatory bowel disease; IBD-U, inflammatory bowel disease-unclassified; IT, Information Technology.

### Quantification of PRO-2 symptoms

To study clinical practice with respect to quantifying symptoms using standardised descriptors, we focused on the PRO-2 items, namely stool frequency (both CD and UC), severity of abdominal pain (CD) and severity of rectal bleeding (UC).

#### Stool frequency

Overall, some form of stool count (per day) was elicited in 76 of 86 eligible consultations (88%). However, evaluation over a clearly defined time interval was rare. For patients with CD, eliciting stool frequency for the previous 24 hours (HBI) occurred in just 3 of 32 relevant consultations (9%) and assessment over 7 days (CDAI) in just one case (3%). For UC/IBD-U, an assessment over 3 days (Mayo Score) was never observed in 44 relevant encounters, whereas a timeframe of 7 days was seen in one case (2%). Within the EHR, rates of recording stool frequency during a standardised timeframe were very low for both forms of IBD. For CD, the rates of recording a 24-hour or 7-day timeframe were, respectively, 4% and 2% (n=253 records). For UC/IBD-U, corresponding rates for 3-day or 7-day assessment periods were 1% in both cases (n=281 records). Interestingly, lack of specification of a precise time period was observed even in consultations where a disease activity score was generated, suggesting such scores were not strictly valid. Of note, stool frequency was specified relative to normal, as defined in the Mayo Score, in just half of consultations where this outcome was elicited or recorded (56% consultations and 49% records).

#### Abdominal pain severity

Pain was verified as being an active symptom in 27 observed consultations of patients with a diagnosis of CD, with severity quantified using standardised descriptors (mild, moderate or severe) in only 15 (55.5%). Non-standard descriptors of severity were used in a further seven (26%), leaving five with no observed discussion of severity (18.5%). Assessment over a standardised timeframe was rare (over last 7 days, as per CDAI, in one case (4%); last 24 hours, as per HBI, in two cases (7%)). There were 160 EHRs of consultations for CD where abdominal pain was noted to be an active symptom, with only 41 (26%) using the standardised descriptors, 71 (44%) using non-standard descriptors and 48 (30%) having no record of severity. None of these records indicating that pain was assessed over a specific time period.

#### Blood in stool

This was an active symptom covered in 20 observed consultations for UC/IBD-U but bleeding severity was quantified using standardised descriptors from the Mayo Score during only six of those encounters (30%) and those from the SCCAI in just seven cases (35%). Similarly in the EHR, recording of quantification of bleeding severity within 134 relevant records was identified, respectively, in 24 for Mayo (18%) and 29 for SCCAI (22%).

### Coverage of outcome sets from the disease activity indices

Complete coverage of all symptom and sign domains required for disease activity indices was rare, although coverage of just the symptom items was more common (table 6). Rates were highest for the symptoms from the HBI for CD (76% of observed consultations, 31% of records) and for the simple partial Mayo Score for UC (46% and 31%, respectively).

**Table 6:** Frequency of eliciting sets of symptoms and signs from relevant disease activity indices for Crohn’s disease and ulcerative colitis during observed consultations and of recording them in the electronic health record

Disease activity index and outcome set	Elicited during observed consultation, n (%)*	Recorded in electronic health record, n (%)*
Crohn’s disease		
Harvey-Bradshaw Index		
Main symptoms (general well-being, number of liquid stools, abdominal pain)	29 (76)	119 (31)
All domains (general well-being, number of liquid stools, abdominal pain, abdominal mass and one or more of the following: eye symptoms, joint symptoms, skin symptoms, mouth ulcers, anal fissures/fistulae or abscesses)	7 (18)	18 (5)
Crohn’s disease activity index		
Main symptoms (general well-being, number of liquid stools, abdominal pain)	29 (76)	119 (31)
All domains (general well-being, number of liquid stools, abdominal pain, weight, abdominal mass and one or more of the following: eye symptoms, joint symptoms, skin symptoms, mouth ulcers, anal fissures/fistulae or abscesses, fever)	3 (8)	7 (2)
Ulcerative colitis or IBD-U		
Simple Clinical Colitis Activity Index		
Main symptoms (general well-being, day stool frequency, night stool frequency, blood in stool, urgency)	14 (29)	38 (10)
All domains (general well-being, day stool frequency, night stool frequency, blood in stool, urgency and one or more of the following: eye symptoms, joint symptoms, skin symptoms)	9 (19)	14 (4)
Partial Mayo Score		
Main symptoms (stool frequency relative to normal, blood in stool)†	22 (46)	125 (31)

The figures indicate whether the relevant items were covered, regardless of the approach to quantifying the outcome.

*Patients with a stoma excluded.

†Includes eliciting or recording stool frequency as being ‘normal’.

Only 7 of the 24 clinicians (three nurses and four doctors) ever collected or recorded a complete disease activity index (HBI or SCCAI). Interestingly, only 4 of 38 (10.5%) clinical records of consultations where biologic therapies were initiated had a disease activity index recorded in the EHR, despite a formal assessment of disease activity being a key performance indicator for the UK Biologics Audit.^19^

As with PRO-2, exploratory models identified no significant patient, practitioner or site level factors associated with eliciting relevant symptom items of selected indices (HBI and SCCAI) within the 102 observed consultations (data not shown). However, in the three-way models of EHRs, the likelihood of finding the main set of symptoms from HBI captured in CD records was independently associated with hospitals with a high degree of digital maturity (OR: 3.09 (95% CI 1.14 to 8.37), online supplemental information S4) and was almost significant for nurse-led consultations (OR: 2.34 (95% CI 0.98 to 5.60)). With regards to records for UC/IBD-U, there was a significant association for nurses recording the main symptoms from SCCAI (OR 20.15 (95% CI 3.82 to 106.33), online supplemental information S5).

### Coverage of outcomes from the complete reference list of symptoms, signs and impacts

Next, we measured the extent to which the presence or absence of our reference list of 37 items were covered systematically during consultations and explored variation in this ‘breadth’ of coverage from one consultation to another.

All the items were elicited during at least one observed encounter, confirming their relevance to IBD practice. Figure 1 shows the relative frequencies of eliciting individual outcomes during observed consultations (A) or recording them within the EHR (B), stratified by diagnosis. ‘General well-being’ was almost invariably mentioned during observed consultations. Most elicited outcomes were symptoms (75%), followed by life impact outcomes (19%) and physical signs (6%). As expected, figure 1 shows that the frequency of eliciting and recording certain individual symptoms was statistically different between the two main forms of IBD, most notably the coverage of blood in stool, mucus in stool and nocturnal stools more frequently in UC. Nevertheless, even these items were not covered at all encounters with UC cases. With respect to CD phenotype, abdominal pain was covered more frequently in consultation records for patients with stricturing as opposed to non-stricturing disease (65% vs 55%; p=0.001), although this still left a third of visits with no explicit record of the presence or absence of abdominal pain.

A median of 12 outcomes (IQR 8–14) were elicited during the 102 observed consultations, with no statistical difference between CD and UC/IBD-U (12 (7–14) vs 12 (9–14)). This represents only one-third of potential outcomes covered in a typical consultation. Remarkably, there was 13-fold variation in the number of outcomes discussed, ranging from 2 to 26.

A median of 8 (IQR 4–11) outcomes were recorded in the EHR following an observed consultation, comprising just under a fifth of the list and there was 21-fold variation between the records (range: 1–21; with a small minority recording none of outcomes on the list, n=3). The mean ‘information loss’ between outcomes discussed and those subsequently captured in the EHR for the same consultation was 3.45 outcomes, with mean 2.37 symptoms not captured in EHR per consultation.

In our large-scale review of 909 EHRs generated by 127 clinicians, there was a median of 7 (IQR: 4–10) outcomes per visit recorded overall, with 7 (4–10) for CD and 6 (4–9) for UC/IBD-U. This constitutes less than a fifth of the pre-specified list. Again, there was substantial variation between consultations (range: 0–23). Furthermore, when we calculated the cumulative count of outcomes recorded for each patient over the preceding year from the observed clinical encounter, this was also relatively low, comprising only a third of pre-specified outcomes (median 13 (9.75–17) over median 3 consecutive clinic visits (2–7)), with over 13-fold variation (range 2–27).

**Figure 2:**
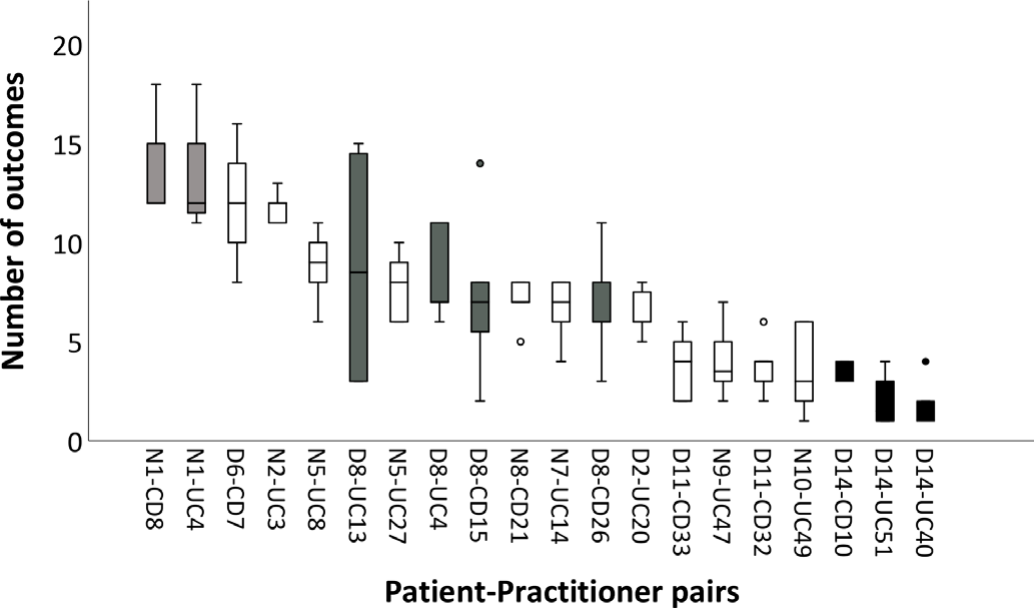
Number of outcomes recorded in the electronic health record for consecutive consultations by the same practitioner with an individual patient. The number of consultation records ranges from 5 to 9 per practitioner–patient pair. Boxplots show median, IQR, minimum–maximum and outlier values of outcomes covered from the pre-specified list. Doctors (D), nurses (N), patients with ulcerative colitis (UC) and those with Crohn’s disease (CD) are numbered. Hence N1-CD8 indicates consecutive consultations of nurse number 1 with Crohn’s disease patient number 8. Boxplots for selected practitioners are coloured. Nurse 1 (light grey) recorded a consistently high median number of outcomes, Doctor 8 (dark grey) showed more variability within patients and Doctor 14 (black) recorded a consistently low median number.

Most clinicians showed a high degree of variability in outcome coverage from case to case. Using the large sample of EHRs, we aggregated data for multiple consultations between the same patient and practitioner. While showing variation within and between such patient–practitioner pairs (figure 2), this also revealed that certain practitioners tended to record a consistently high (Nurse 1), medium (Doctor 8) or low (Doctor 14) number of outcomes from case to case, pointing to potential systematic differences in their practice.

### Coverage of three or more symptoms in a standardised sequence of questions

To further explore whether each observed clinician asked questions in a standardised sequence, we identified whether there was evidence for eliciting any group of three or more symptoms in a fixed order in more than one observed consultation. The use of an ordered checklist was observed in only 7 (14%) consultations for CD and 16 (31%) for UC, all of which were conducted by nurses. The standardised sequences consisted of a mean of 6 (range: 3–11) questions. Median number of outcomes elicited in consultations where a standardised sequence of questions was used was higher compared with consultation without a checklist approach (14 vs 11, p<0.001).

### Practitioners’ views on the variation in outcome coverage in routine practice

To further interpret variability in outcome coverage demonstrated by descriptive analyses, we used qualitative approaches to explore characteristics of personal practices that were not amenable to quantitative enquiry. During observations of IBD consultations, we witnessed wide variation in time, structure and content of encounters, and observed a range of individualised assessments of health status conducted by practitioners. In interviews, clinicians commonly described the need to tailor health status assessment to an individual patient, as opposed to adopting a standardised approach. However, a minority of participants advocated the use of a personal ‘checklist’ when eliciting symptoms to ensure all relevant items were covered.

Clinician interviewees identified a range of patient, clinician and hospital factors contributing to variation in outcome coverage which are summarised in online supplemental information S6. Interviewees attributed variation to their initial impressions of overall patient’s health and disease activity (Quotes 1–2), treatment compliance (Q3), availability of objective tests to indicate active or inactive disease (Q4) or perceptions of patient’s personality (Q5–7). Practitioners’ training background, skills and experience were also reported as important sources of variation (Q8–12). Many practitioners described that specialist nurses were more thorough (or less selective) and systematic in their assessments, and therefore actively covered more outcomes than doctors (Q11–12). Moreover, clinicians described specialist IBD clinics and local hospital EHR as helping to support the collection of consistent, structured outcomes as these served as a prompt and facilitated capture (Q16–19).

## Discussion

Our study demonstrated that coverage of a basic minimum symptom set (PRO-2 pairs) was a common but not an invariable part of routine IBD practice, that capture of complete disease activity indices was relatively rare and that the quantification of individual outcomes did not adhere closely to standardised descriptors. There was substantial inter-individual and intra-individual variation among practitioners in eliciting IBD outcomes and a significant deficit in the elicited information being then recorded in the EHR. Clinicians tailored health status evaluations to individual patients, expressed preference for personalised assessments and defined a range of patient, clinician and hospital factors contributing to variation in outcome coverage.

While some degree of variation was expected due to heterogeneity of clinical characteristics from one case to the next, the magnitude of inter-practitioner variation is very large and appears unexplained by legitimate clinical factors. Our findings are consistent with previously described heterogeneity in the clinical outcomes and quality measures found in routine EHR,^29^ and the low rates of standardised disease activity information captured by the National Audit of Biological Therapies in the UK.^19^ Poor standardisation in practice may partly reflect a current lack of consensus on the most suitable CLIN-ROs and PROs for clinical trials.^5–7^ It is noteworthy in the case of IBD that explicit recommendations for ‘minimum’ clinical outcome sets are largely lacking from specialist guidelines of the American College of Gastroenterology,^30 31^ European Crohn’s and Colitis Organisation^32^ and British Society of Gastroenterology.^25^ Standards for outcome assessment are not covered by current UK and US quality standards.^33 34^ We believe our findings are in keeping with lack of standardisation of outcome assessment in other disease areas, as little research exists on variation in capturing outcomes in other conditions, despite the potential major implications for inequality in decision-making and health outcomes.

However, it must be noted that the benefits of greater standardisation and impact on individual health outcomes remain unknown. There is no current evidence to suggest that adoption of a uniform, standardised approach to outcome assessment delivers better results for patients with IBD than more flexible, unstructured practice. Future research is needed to study inequalities associated with variation in clinical practice, including impact on therapeutic decision-making, treatment access and health outcomes. If standardisation of outcome assessment is to be embedded in routine practice, education, training and regulatory oversight will be needed to transform clinicians’ workflows, as well as additional resource and IT capabilities to enable outcome capture.

Our study showed that IBD specialist nurses recorded outcome sets more frequently than doctors, and demonstrated greater efforts to standardise routine assessments of health status. This highlights an important role of IBD nurses in capturing standardised outcomes as part of the care delivery process, and suggests that their contribution to routine data collection could be further used.

Our observations suggest that the use of traditional indices and scoring systems are seldom part of the process of making individual patient decisions in routine care. This highlights the divergence between clinical trials and practice. It is recognised that Randomised Controlled Trials do not reflect the selection of patients, nor the approach to judging treatment effectiveness in day-to-day practice.^35^ This highlights the need for a debate between researchers, clinical experts and patients on optimal ways to develop and implement COS both for clinical trials and practice. Future pragmatic studies are needed to address the evidence gap between traditional clinical trials and the process of routine decision-making. In the interim, there is an unmet need for an explicit set of minimum standards for IBD outcome assessment in routine settings in the UK and worldwide, and audit and quality improvement initiatives to evaluate any benefits of associated changes to clinical practice. Future research is needed to study inequalities associated with variation in clinical practice, including impact on therapeutic decision-making, treatment access and health outcomes.

Our findings also reveal major challenges for utilising routinely recorded outcome data for clinical trials and observational research. Lack of routine collection of standardised clinician-reported end-points as part of the care delivery process is a barrier to undertaking large scale analysis of real-world patient outcomes in the UK, including initiatives such as UK IBD Registry,^23^ Health Data Research UK IBD Hub^36^ and IBD BioResource.^37^ Leveraging routinely captured outcome data at scale from real-world settings may require computational methods to interrogate varied terminologies and unstructured data. Our present work provides a starting point for building a lexicon of terms and phrases to support Natural Language Processing approaches.^38^

PRO measures are increasingly seen as a practical alternative to reliance on composite activity measures generated by clinicians. This is particularly relevant in the face of the current COVID-19 pandemic which has disrupted traditional face-to-face care and enforced the move to remote consultations, with reduced access and/or willingness to undergo objective testing. Hence clinical assessments have become even more valuable, and adoption of virtual consulting and new models of care have been accelerated.^39^ The COVID-19 pandemic has also highlighted the need for better solutions for remote disease monitoring, and investment in programmes, technologies and infrastructure to allow electronic capture of PRO measures and integration of patient-reported data into operational records.

Our study has some inevitable limitations in terms of generalisability. The scope was limited to six centres and one region of England and we observed only 102 consultations directly. Undertaking structured observations and analysis of ‘live’ consultations is a resource-intensive exercise. However, we included a range of sites (from smaller district general hospitals to tertiary centres) and a wide spectrum of practitioners. Given the high degree of practice variation demonstrated, we believe it is unlikely that such findings are confined to the centres studied. On the contrary, our results are even more noteworthy given that clinicians volunteered to be observed by a researcher, and may have adopted a more structured approach than their usual practice due to the Hawthorne Effect from direct observation.

In conclusion, we have shown substantial variability in the breadth, depth and quantification of IBD clinical outcomes during routine clinical assessments. Evidence-based policies, education, quality standards and audit are needed to define and address unwarranted variations in outcome assessment. Future efforts are required to converge clinical IBD activity assessments between clinical trials and practice. Direct capture of PROs using validated instruments is likely to provide a more feasible approach to capturing standardised outcomes as part of the care delivery process.

## Data Availability

Data are available upon reasonable request. The data that support the findings of this study are available from the corresponding author upon reasonable request.
