# Supplementary material for: Heterogeneity in outcome assessment for inflammatory bowel disease in routine clinical practice: a mixed-methods study in a sample of English hospitals
Source: BMJ Open. 2021 Dec 30;11(12):e056413. doi: 10.1136/bmjopen-2021-056413 (PMC8719195; doi:10.1136/bmjopen-2021-056413)
Supplement: Supplementary data [file bmjopen-2021-056413supp001.pdf]

Supplementary Information S1

List of inflammatory bowel disease outcomes and their sources

	Clinician-reported outcomes (ClinRO)						Patient-reported outcomes (PRO)			
	Crohn's disease			Ulcerative colitis			Crohn's disease		Ulcerative colitis	
**Outcomes**	**CDAI**	**HBI**	Other ClinROs[1]	**MCS**	**SCCAI**	Other ClinROs[1]	PRO2 PRO3	Other PROs[2]	PRO2 PRO3	Other PROs[2]
**SYMPTOMS**										
**General well-being**	✓	✓			✓	✓	✓	✓	✓	✓
**Stool frequency**	✓	✓	✓	✓	✓	✓	✓	✓	✓	✓
**Stool frequency at night**					✓	✓				
**Liquid/soft stools**	✓	✓	✓			✓		✓		✓
**Number of liquid stools**	✓	✓	✓			✓	✓	✓		✓
**Abdominal pain**	✓	✓	✓			✓	✓	✓		✓
**Blood in stool**				✓	✓	✓		✓	✓	✓
**Urgency**					✓			✓		✓
Mucus in bowel movements								✓		✓
Faecal incontinence					✓	✓		✓		✓
Abdominal bloating								✓		✓
Flatulence								✓		✓
Nausea or vomiting								✓		✓
Appetite								✓		✓
Fatigue								✓		✓
Bodily pain								✓		✓
Perianal discharge			✓							
Perianal pain			✓							
**Eye symptoms**	✓	✓	✓		✓	✓				
**Joint pain**	✓	✓	✓		✓	✓				
**Skin symptoms**	✓	✓	✓		✓	✓				
**Mouth ulcers**	✓	✓	✓			✓				
**SIGNS**										
Pulse						✓				
**Fever**	✓		✓			✓				
**Body weight**	✓		✓					✓		✓
**Abdominal mass**	✓	✓	✓							

**Anal fissures, fistulae or abscesses**	✓	✓	✓							
**IMPACTS**										
Perceived health status								✓		✓
Physical function								✓		✓
Role function								✓		✓
Social function								✓		✓
Emotional function								✓		✓
Sleep function								✓		✓
Sexual function			✓					✓		✓
Perception of disease control								✓		✓
Perceived treatment effectiveness								✓		✓
Side effects of IBD treatment								✓		✓

CDAI – Crohn's Disease Activity Index

HBI – Harvey-Bradshaw Index

MCS – Mayo Clinic Score [or PMS – Partial Mayo Score]

SCCAI – Simple Colitis Clinical Activity Index

1. Other ClinROs – Perianal Disease Activity Index, Dutch or Van Hees Activity Index, Ulcerative Colitis Disease Activity Index (Sutherland Index), Clinical Activity Index (Rachmilewitz Index), Truelove and Witts Index

PRO-2 – Two Item Patient Reported Outcome

PRO-3 – Three Item Patient Reported Outcome

2. Other PROs – the Inflammatory Bowel Disease Questionnaire, 36-Item Short Form Survey, EQ5D EuroQol Questionnaire, IBD-Control-8, Crohn's Ulcerative Colitis Questionnaire–8, IBD Patient-Reported Treatment Impact Survey, The Manitoba Inflammatory Bowel Disease Index

Items from the main disease activity indices (CDAI, HBI, Mayo score and SCCAI) are highlighted in red.

Supplementary Information S2

Interview Topic Guide

This topic guide outlines the questions and prompts that were used during the interviews. It was adapted as the study proceeded according to participant's responses and the emerging analysis.

Introduction:

- Explain the aims of the study
- Confirm consent to audio-record interview and use quotes in the write up of the study

Discussion:

1. **Assessment of clinical outcomes**
    - How do you structure your consultations with patients in IBD clinic?
    - What symptoms and signs are most important to you when assessing IBD activity? What key symptoms do you collect?
    - What symptom information do you collect when making decisions to initiate or modify anti-inflammatory therapy in IBD?
    - What are your views on the content and structure of current medical records in IBD?
    - [Discuss relevant aspects of clinical practice observed during consultations]
2. **Variation in outcome assessment**
    - Do you follow a pattern of questions when assessing IBD activity in clinic?
    - Is there any variation in symptom information you elicit from patients?
    - Are you consistent in assessing signs and symptoms in consultations? If not, how does your approach change and when?
    - In your view, what factors influence symptom information you collect? [Prompts - disease severity / patient preferences / EHR]

3. **Structured outcomes**
    - Do you collect any structured clinical outcomes in IBD clinic? [Disease activity indices /PROMs] Why?
    - What is your opinion on clinical disease activity indices? Do you find them useful in day to day practice?
    - What impact does collecting disease activity scores have on consultations?
    - Have you experienced any difficulties in collecting or recording disease activity indices? Have you thought of any solutions?
4. **Standardised health status assessments**
    - What is your opinion on standardised health records in IBD? [UK IBD Registry / local EHR datasets]
    - Do you / would you use them in clinic? How would they fit in your clinic flow?
    - Do you have access to any structured data relating to your patients?
    - Do you see any potential benefits in collecting structured clinical information? [Prompts – audit / research]
    - Do you see any problems in using standardised data templates in clinic? [Prompts – loss of patient-centredness / time pressure] Could you share any potential solutions?

5. **ICHOM Patient-centred Outcomes for IBD**
    - What is your opinion on the ICHOM outcome set?
    - What outcomes from the ICHOM set do you collect in clinic and why?
    - How would you capture these outcomes in routine clinic?
    - Are there any barriers and potential solutions? [Feasibility]

Is there anything else that wasn't covered that you think is important?
……………………………………………………………………………………………
Close interview

Supplementary Information S3

Flowchart of data collection and analysis

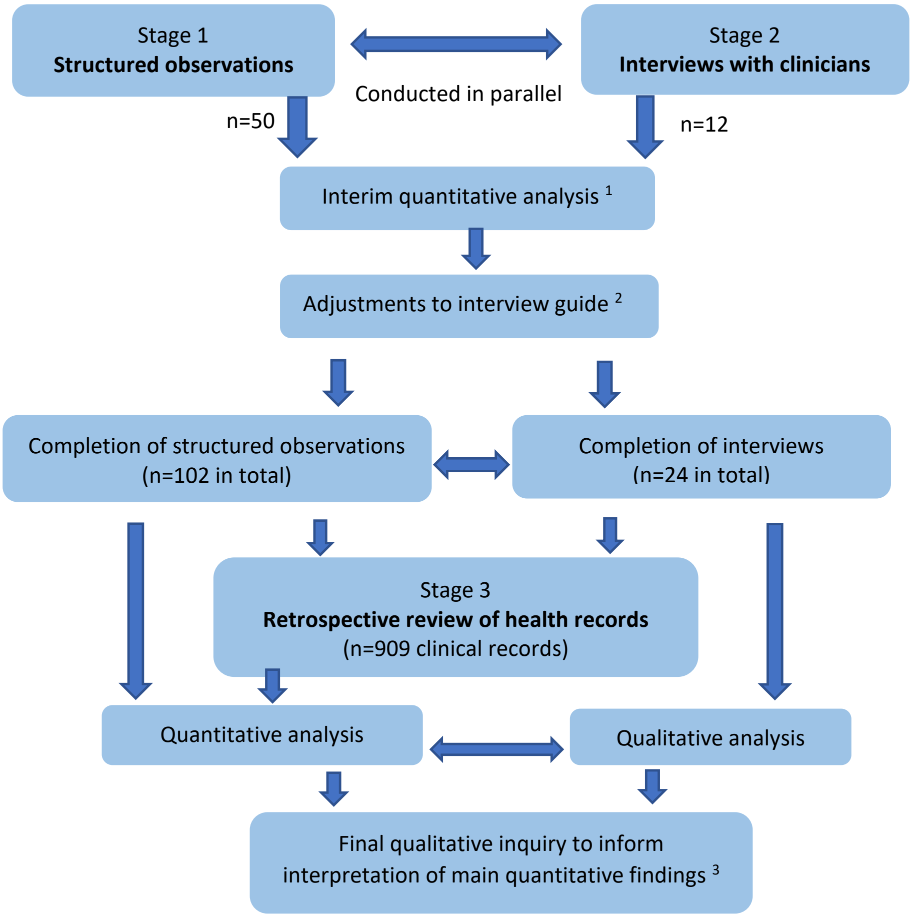

1- Coverage of PRO- symptom pairs and disease activity indices in observed consultations
2- Focus on individualised health status assessments, and factors associated with variation in outcome coverage
3- Themes relevant to variation in outcome coverage between and within patients and clinicians, and views on standardisation of outcome collection in routine care

Supplementary Information S4

Factors associated with eliciting or recording of symptom items of Harvey Bradshaw Index (HBI) in consultations for Crohn's disease. Symptom items were general wellbeing, number of liquid stool and abdominal pain. Random effects binary logistic regression models for selected patient, practitioner and site characteristics. For observed consultations, there were no significant associations for eliciting the relevant symptom items. Within the electronic health record, the likelihood of finding the appropriate symptom items recorded was independently associated with hospitals with more mature IT systems (three-fold; p=0.026). OR = odds ratio; CI = Confidence Intervals; p = p-value.

	**Symptom items of HBI recorded** (Review of electronic health records) n=384			
	Univariate analysis		Multivariable analysis	
	OR (95%CI)	*p*	OR (95%CI)	*p*
**Patient factor**				
Female gender	2.20 (1.08-4.51)	0.030	2.17 (0.99-4.76)	0.053
Age	0.98 (0.96-1.01)	0.190	0.99 (0.97-1.02)	0.483
Previous Surgery	0.71 (0.35-1.44)	0.337	0.60 (0.25-1.43)	0.247
Extraintestinal manifestations	1.52 (0.64-3.63)	0.344	1.42 (0.58-3.43)	0.441
Current immunosuppressive therapy	2.82 (1.28-6.20)	0.010	2.15 (0.94-4.91)	0.071
Disease duration	1.01 (0.98-1.04)	0.613	1.01 (0.97-1.05)	0.704
**Practitioner factor**				
Nurse consultations	2.51 (1.03-6.15)	0.004	2.34 (0.98-5.60)	0.056
**Hospital IT factor**				
Global Digital Exemplars	5.24 (1.93-14.22)	0.001	3.09 (1.14-8.37)	0.026

Supplementary Information S5

Factors associated with eliciting or recording of symptom items from Simple Clinical Colitis Activity Index (SCCAI) in consultations for ulcerative colitis and IBD-U. Symptom items were general wellbeing, day stool frequency, night stool frequency, blood in stool and urgency. Random effects binary logistic regression models for selected patient, practitioner and site characteristics. For observed consultations, there were no significant associations for eliciting the relevant symptom items. Within the electronic health record, the likelihood of finding the appropriate symptoms was independently associated with nurse-led visits (twenty-fold; p<0.001). OR = odds ratio; CI = Confidence Intervals; p = p-value.

	**SCCAI symptom items recorded** (Review of electronic health records) n=398			
	Univariate analysis		Multivariable analysis	
	OR (95%CI)	*p*	OR (95%CI)	*p*
**Patient factor**				
Female gender	0.61 (0.25-1.48)	0.273	0.74 (0.29-1.86)	0.519
Age	0.99 (0.96-1.02)	0.582	0.98 (0.95-1.02)	0.362
Extraintestinal manifestations	0.39 (0.06-2.35)	0.303	0.52 (0.09-2.95)	0.461
Current immunosuppressive therapy	0.55 (0.19-1.61)	0.275	0.47 (0.16-1.37)	0.167
Disease duration	0.99 (0.96-1.03)	0.708	1.00 (0.97-1.04)	0.850
**Practitioner factor**				
Nurse consultations	20.38 (3.60-115.23)	0.001	20.15 (3.82-106.33)	<0.001
**Hospital IT factor**				
Global Digital Exemplars	0.72 (0.11-4.83)	0.738	0.73 (0.14-3.69)	0.701

Supplementary Information S6

Practitioner's views on the sources of variation in outcome coverage for IBD in routine practice. Findings from one-to-one interviews with 24 clinicians across six hospitals in England. D = Doctors, numbered 1 to 14; N = Nurse Specialists, numbered 1 to 10.

Themes	Interview quotes
**Patient factors**	
There is variation in the selection and breadth of outcomes covered ***within*** individual patients<br><br>*Potential sources of variation are: perception of disease activity, course and control, treatment compliance*	(1) *When people are acutely flaring […], and that is the main reason why the patient comes back, symptoms will take the priority of the consultation* [D14]<br><br>(2) *If they say they feel well, they are on medications, there are no issues, bloods are all ok, and they are happy with things, I might not probe in as much [D11]*<br><br>(3) *Some patients will say they take medications, but TGN [thioguanine nucleotide] levels are always sub therapeutic. You do worry that there is an element of non-compliance. In those circumstances, I tend to then ask the whole lot. But when they are already established well on the therapies, there's evidence that we've checked their levels and that they are taking it regularly, […] I'm not as strict* [D11]<br><br>(4) *If all objective markers are normal, I might just make it quicker. I still […] have questions, but it might be a quicker process of asking them and moving on from them* [D6]
There is variation in the selection and breadth of outcomes covered ***between*** patients<br><br>*Potential sources of variation are: personality types, individual concerns, psychological morbidity*	(5) *Some patients tend to over report their symptoms and some under report, just by their personality type* [D2]<br><br>(6) *Patients that I find quite difficult […] to assess fully are those with psychological problems. The majority of their clinic consultation is spent on looking at their coping mechanisms, and how they are managing, and very little time is spent on how they are physically* [N1]<br><br>(7) *If I have known them before and I know that they are worried about cancer or whatever, there are certain things that I focus on more with some patients* [N7]
**Clinician factors**	
There is variation in the selection and breadth of outcomes covered ***between*** clinicians<br><br>*Potential sources of variation are:*	(8) *Each of us work to our own template. No one has come to me and said, this is how we expect our letters to be dictated […] I have been doing it my own way* [D13]<br><br>(9) *Different people have different styles and you learn how colleagues of yours assess patients, and some people are very detailed, others are not so detailed* [D1]

individual consultation styles and record-keeping, role and experience	(10) *There is … variability depending on your interest, knowledge, expertise* [D11]<br><br>(11) *Nursing perspective is very different compared to consultant perspective. So nurses will often follow a set criteria whereas I will often be driven by what the patient is like* [D3]<br><br>(12) *Nurses are much better at data collection […] As a consultant, you can judge, you can say, I know things are ok, I know things are right based on this, and I don't necessarily have to have a form to follow* [D9]
There is variation in the selection and breadth of outcomes covered ***within*** individual clinicians<br><br>*Potential sources of variation are: external factors, time pressures, clinicians' preferences, contextual factors*	(13) *You can have clinics where there are the adequate numbers [of clinicians] or the ratio is really good of patients to consultants. Therefore, good quality data is collected. You then have a really massively overbooked clinic and then that data quality reduces dramatically because there's just not enough time to fit all that in* [D5]<br><br>(14) *If you think something's important to follow through, you will follow-through, but I have no doubts there will always be inconsistencies as much as every person is their own unique being* [D11]<br><br>(15) *Sometimes it depends on how you feel. That's got a lot to do with it. There are external pressures involved with a clinic* [N9]
**Hospital factors**	
Recording of outcomes is facilitated in sites with mature and user-friendly EHR systems	(16) *[Electronic IBD patient record] is a good prompt to gather information [...] you've got it in front of you and you have to pick one of those options* [D6]<br><br>(17) *Those electronic systems all have their varying degree of how much attention you need to give them. […] We're quite lucky that we have our [EHR] system where it makes it easier for us to record disease activity* [D5]
Outcome coverage is more focused and consistent in specialist IBD clinics	(18) *If it's a an IBD clinic, I'm more consistent than a general clinic* [N9]<br><br>(19) *In a specialist clinic you have the clinic structured so that what needs covered is covered […], rather than having to try and do it after you have seen the patient* [D8]
